# Editorial: Regulation of Soluble Immune Mediators by Non-Coding RNAs

**DOI:** 10.3389/fimmu.2020.607222

**Published:** 2020-10-15

**Authors:** Daniela Bosisio, Flavia Bazzoni

**Affiliations:** ^1^Department of Molecular and Translational Medicine, University of Brescia, Brescia, Italy; ^2^Department of Medicine, Division of General Pathology, University of Verona, Verona, Italy

**Keywords:** microRNAs, Y-RNAs, lncRNAs, toll-like receptors (TLRs), extracellular vesicles (EVs), ribonucleoproteins, autoimmunity, endotoxin tolerance

Non-coding RNAs (ncRNAs), defined as transcripts that do not encode proteins, are known since long time for their role in translation (i.e. transfer RNAs, ribosomal RNAs) and in splicing events (i.e. small nuclear and small nucleolar RNAs). However, only recently, the revolutionary advances in deep sequencing technology brought to light several new classes of ncRNA, classified according to their length into “short” ncRNAs (<200 nucleotides, that includes piwi-associated RNAs, endogenous short-interfering RNAs, microRNAs, Y-RNAs and others), and “long” ncRNAs (lncRNAs, >200 nucleotides) (1).

Cytokines are crucial soluble messengers of the immune system that regulate and sustain inflammation and immunity. Cytokine expression is tightly regulated, reflecting the need of the immune system to tailor the magnitude and duration of its responses to induce pathogen clearance, but not tissue damage. Thus, understanding cytokine regulation is crucial to gain insight and eventually manipulate undesired immune responses.

In this Research Topic, 53 authors contributed 11 articles touching on many of the combined roles of ncRNAs on the production of cytokines and their consequential effect on cytokine-related functional outputs, as well as inflammatory/autoimmune pathologies.

## Immune Regulation by Intracellular ncRNAs

Other than the size limit of 200 nt and a lack of protein-coding potential, the sole other common feature of all ncRNAs consists in being functionally implicated in gene regulatory processes. This is achieved via a multitude of mechanisms, ranging from promoter-specific repression, transcriptional activation, epigenetic remodeling, or post-transcriptional gene regulation such as translational blockade and/or activation (1). Based on these features, the regulatory functions of ncRNAs are recognized to be involved in virtually all homeostatic, developmental and reactive pathways and systems, including the immune response (2, 3).

**Figure 1:**
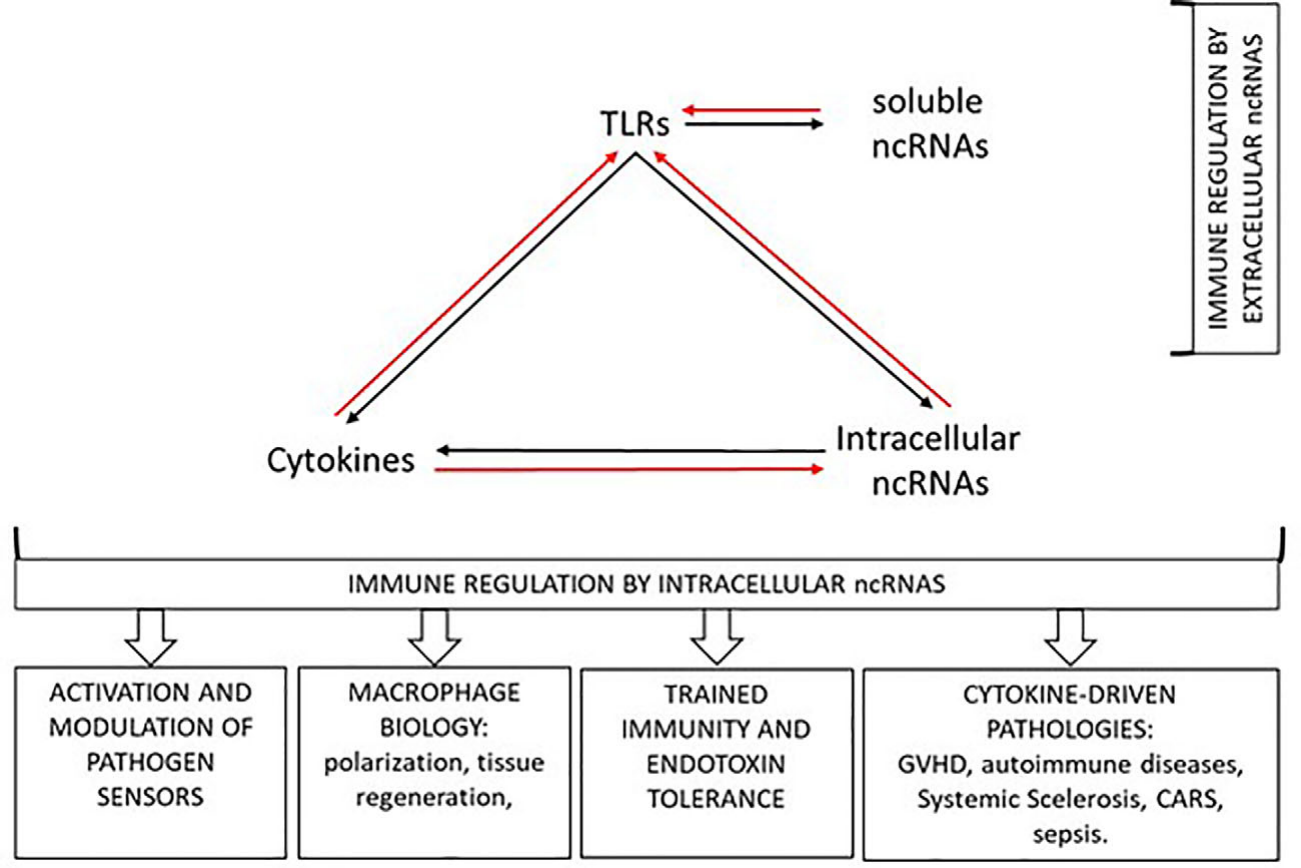
Multifaceted crosstalk between cell activation, ncRNAs, and cytokine expression (see text).

Among ncRNAs, microRNAs (miRNAs) currently represent the best characterized post-transcriptional regulators of cytokine production. In this Research Topic, Garavelli et al. review the literature concerning regulation of adaptive cytokines by miRNAs, while Salvi et al. concentrate on miRNA-dependent regulation of cytokines that are hallmarks of autoimmune diseases. The emerging picture is quite complicated for several reasons. First, the final effect on cytokine levels may derive by direct regulation of cytokine RNA or by the modulation of cytokine inducers or repressors. In addition, as these authors underline, the coordinated induction and modulation of tens of miRNAs may be required to efficiently affect the components of a genetic network. Thus, it is crucial to rapidly break away from the musty assumption “one miRNA, one cytokine” to boldly embrace the “rheostat” function of miRNAs and to be able to frame the mechanistic miRNA regulation in process-specific contexts. One example of such “integrated view” is beginning to emerge in the multifaceted crosstalk between cell activation, ncRNAs and cytokine expression (**Figure 1**).

Toll-like receptors (TLRs) are key pathogen receptors of the innate immune system. The first miRNA described as induced following TLR activation and controlling TLR signaling through a negative feedback regulation loop dates back to 2006 (4). Since then, our knowledge on the miRNAs induced upon cell activation downstream TLRs and on the mechanisms through which these miRNAs feedback modulate immune cell responses has grown enormously. More recently, a number of lncRNAs have been included among the non-coding transcripts with regulatory functions in the TLR signaling pathway. Three reviews of this Research Topic are focused on the role of ncRNA in immune cell activation downstream TLRs and on the pathologies driven by dysregulation of the TLR-induced responses. Bayraktar et al. summarize the potential role of miRNAs in regulation of gene expression and TLR signaling, with a focus on the ability of miRNAs to act as endogenous ligands of specific TLRs and trigger the downstream immune response (see further). The complexity of this highly regulated network of ncRNAs in macrophage biology is further discussed by Curtale et al., with particular emphasis on the role of miRNAs in macrophage heterogeneity and plasticity in response to environmental cues, bacterial infection, tissue regeneration and endotoxin tolerance. Further studies on TLR-induced miRNAs and lncRNAs in the regulation of endotoxin tolerance are comprehensively reviewed by Vergadi et al., and their impact in the context of innate immune tolerance and of sepsis is discussed. Together with the abovementioned reviews, an original article by Mariotti et al. identifies a role for a lncRNA (namely NRIR) in the expression of type I Interferon Stimulated Genes (ISGs) in monocytes downstream TLR4 activation. Remarkably, this study highlights that aberrant expression on NRIR can be involved in the dysregulation of the innate immune system linked to the development of Systemic Sclerosis.

## Immune Regulation and Biomarker Function of Extracellular ncRNAs

The picture of immune regulation by ncRNAs is further complicated by their travelling in extracellular spaces, either encapsulated in extracellular vesicles (EVs) or associated to macromolecular structures such as ribonucleoproteins and lipoprotein particles. Despite the function of most extracellular ncRNAs remains largely elusive, they are in the scientific limelight because of a possible role as regulators of intercellular communications as well as a tremendous potential as non-invasive biomarkers for multiple disorders, including pathologies of the immune system (5).

Such burning interest well reflects in our Research Topic, where five contributions deal with different aspects of extracellular ncRNAs biology. Turchinovitch et al. provide a state-of-the-art overview of the transcriptome of EV-associated RNAs, where miRNAs represent the most intensively studied component and, at the same time, the minority of all EV-enclosed RNAs. One prominent class of EV-associated extracellular RNAs involved in a range of immune-mediated processes are the Y-RNAs, discussed here by Driedonks and Nolte-’t Hoen. Both these reviews also address some technical challenges associated with obtaining pure EVs and deep sequencing of the EV-associated RNAs, as well as in assessing whether extracellular ncRNAs are contained in ribonucleoprotein complexes or EVs. These technical aspects are crucial to overcome the frequently observed inconsistency in the identification and quantification of extracellular ncRNAs, which currently impairs our capacity to use them as reliable biomarkers.

Both these reviews, as well as other contributions (Garavelli et al.; Salvi et al.; Bayraktar et al.; Zitzer et al.), also converge in highlighting a role for extracellular ncRNAs as ligands of RNA sensors, TLRs in particular. This function was recently demonstrated for EV-associated miRNAs (6) and may play a role in inducing unwanted inflammation and tissue damage, as reviewed here by Zitzer et al. In this regard, based on the largely sequence-independent impact of nucleic acids on the TLRs (6), Turchinovitch et al. and Driedonks and Nolte-’t Hoen point out the strong possibility that more abundant non-miRNA classes could significantly contribute to such activation.

## Therapeuting Exploitation of ncRNAs

It is not surprising to find deregulated ncRNAs as major contributors of cytokine-driven pathologies ranging from acute graft-versus-host disease, autoimmune diseases, Systemic Sclerosis, compensatory Anti-inflammatory Response Syndrome (CARS), endotoxin tolerance and sepsis, as showcased in this Research Topic.

The other side of this same coin would consist of therapeutical exploitation of ncRNAs. Our current lack of a full understanding of their biology and of the intricate network of interactions with the human genome, transcriptome and proteome restrains the translation of such strategies into the clinical use. In addition, a number of specific challenges associated with ncRNA targeting still need to be addressed, such as predicting possible off-target effects and toxicity, improving stability and optimizing the delivery systems (7). In this regard, the original contribution by Macleod et al. focuses on the prevention of paradoxic inflammation following topical delivery of RNA aptamers to treat inflammatory skin diseases.

Despite these knots to be solved, a number of miRNA-based therapeutic tools, mainly for cancer management, entered the clinical trial in the last 5 years (8). Here, one original work by Kim et al. propose miR-135-5p as a target for the development of anti-allergic drugs based on its capability to interact with p62, a selective receptor of autophagy.

## Concluding Remarks

As a result of almost two decades of extensive investigations, nowadays miRNAs can be listed among the soluble mediators of the immune response. In addition, more ncRNAs promise to hold the scene in the near future and for a long time. As we gain more knowledge about the exciting properties of ncRNAs, however, we also get aware of the intricacy of the emerging picture. **Figure 1** schematizes how the bidirectional interplay between cytokine-modulated ncRNAs expression and, in turn, ncRNAs-driven control of cytokine expression and production is further complicated by the recent discovery of the ability of ncRNAs to trigger activation of specific immune receptors. The scrupulous untangling of this intricate web will allow to fully exploit he tremendous potential of ncRNAs as biomarkers and therapeutic tools to safely redirect undesired immune responses.

## Author Contributions

DB and FB were editors of this Research Topic and wrote this editorial jointly. All authors contributed to the article and approved the submitted version.

## Funding

DB and FB are supported by Ministero dell’Università e della Ricerca (PRIN 2017), University of Brescia (Fondi Locali 2019), University of Verona-Joint Project (project JPVR17WCBR) and Fondo Unico per la ricerca (FUR).

## Conflict of Interest

The authors declare that the research was conducted in the absence of any commercial or financial relationships that could be construed as a potential conflict of interest.

## References

[1] Zhang P Wu W Chen Q Chen M Non-Coding RNAs and their Integrated Networks. J Integr Bioinform (2019) 16(3):20190027. 10.1515/jib-2019-0027 PMC6798851 31301674

[2] Chen YG Satpathy AT Chang HY Gene regulation in the immune system by long noncoding RNAs. Nat Immunol (2017) 18(9):962–72. 10.1038/ni.3771 PMC9830650 28829444

[3] Mehta A Baltimore D MicroRNAs as regulatory elements in immune system logic. Nat Rev Immunol (2016) 16(5):279–94. 10.1038/nri.2016.40 27121651

[4] Taganov KD Boldin MP Chang KJ Baltimore D NF-kappaB-dependent induction of microRNA miR-146, an inhibitor targeted to signaling proteins of innate immune responses. Proc Natl Acad Sci USA (2006) 103(33):12481–6. 10.1073/pnas.0605298103 PMC1567904 16885212

[5] Murillo OD Thistlethwaite W Rozowsky J Subramanian SL Lucero R Shah N exRNA Atlas Analysis Reveals Distinct Extracellular RNA Cargo Types and Their Carriers Present across Human Biofluids. Cell. (2019) 177(2):463–77.e15. 10.1016/j.cell.2019.02.018 30951672 PMC6616370

[6] Bosisio D Gianello V Salvi V Sozzani S Extracellular miRNAs as activators of innate immune receptors. Cancer Lett (2019) 452:59–65. 10.1016/j.canlet.2019.03.021 30910591

[7] Christopher AF Kaur RP Kaur G Kaur A Gupta V Bansal P MicroRNA therapeutics: Discovering novel targets and developing specific therapy. Perspect Clin Res (2016) 7:68–74. 10.4103/2229-3485.179431 27141472 PMC4840794

[8] Shah MY Ferrajoli A Sood AK Lopez-Berestein G Calin GA MicroRNA therapeutics in cancer - an emerging concept. EBioMedicine (2016) 12:34–42. 10.1016/j.ebiom.2016.09.017 27720213 PMC5078622

